# Library-free data-independent acquisition mass spectrometry enables comprehensive coverage of the cyanobacterial proteome

**DOI:** 10.1093/plphys/kiaf334

**Published:** 2025-08-12

**Authors:** David A Russo, Felix R Schneidmadel, Julie A Z Zedler

**Affiliations:** Bioorganic Analytics, Institute for Inorganic and Analytical Chemistry, Friedrich Schiller University Jena, 07743 Jena, Germany; Functional Proteomics, Jena University Hospital, 07747 Jena, Germany; Synthetic Biology of Photosynthetic Organisms, Matthias Schleiden Institute for Genetics, Bioinformatics and Molecular Botany, Friedrich Schiller University Jena, 07743 Jena, Germany

## Abstract

Cyanobacteria have played a leading role in elucidating the fundamental mechanisms behind oxygenic photosynthesis, carbon fixation, the circadian clock, and phototaxis. Such molecular processes rely on proteins at their core. Thus, proteomics has become an indispensable tool in building our understanding of these processes. Amongst the proteomic approaches used, “shotgun proteomics”, where complex protein mixtures are enzymatically digested into peptides and analyzed by liquid chromatography–mass spectrometry, has become the go-to technique for whole-proteome analysis. In this study, we introduce shotgun workflows that excel in speed, throughput, and sensitivity, and allow an in-depth description of the cyanobacterial proteome. The main features of these workflows are the improvement of sample cleanup and digestion through single-pot solid phase-enhanced sample preparation (SP3), the adoption of a previously validated trifluoroacetic acid lysis strategy, and the application of library-free data-independent acquisition. Using the established model organism *Synechococcus elongatus* PCC 7942, we show that these workflows exhibit high quantitative reproducibility and enable the detection of 83% to 85% of all open reading frames, the greatest single-shot coverage achieved so far for a cyanobacterium. These workflows require only a couple of hours of hands-on time and should be applicable to most, if not all, cyanobacterial species. Together with the rapid advancements in mass spectrometry technologies, this work has the potential to accelerate cyanobacterial proteomics.

## Teaser

Single-pot solid phase-enhanced sample preparation (SP3) and trifluoroacetic acid workflows open the door to high-throughput quantitative proteomics in cyanobacteria.

## Introduction

Cyanobacteria are morphologically diverse photosynthetic prokaryotes that inhabit nearly every environment on Earth. They have played a key role in elucidating the mechanisms underlying photosynthesis and carbon metabolism (Lucius and Hagemann 2024; Zedler 2024) due to their ancestral connection to the modern chloroplast. Cyanobacteria have also become models to study other key phenomena such as the circadian clock and phototactic motility, and microbial workhorses for whole cell catalysis and the production of bulk and fine chemicals (Cohen and Golden 2015; Schuergers et al. 2017; Russo et al. 2019; Santos-Merino et al. 2019). Amongst the technologies that have allowed us to study cyanobacteria, proteomics has proven to be a valuable tool in expanding our understanding of their cellular processes (extensively reviewed in Srivastava et al. 2024). The early days of cyanobacterial proteomics relied heavily on the use of single or multidimensional gel electrophoresis. However, with the development of high-performance liquid chromatography (LC) and advances in mass spectrometers, researchers increasingly moved away from gel proteomics to adopt “shotgun proteomics” (Ow and Wright 2009). Shotgun proteomics uses LC–MS to identify and quantify proteoforms using peptide mixtures derived from enzymatic digestion of whole proteomes. To date, it remains the gold standard of bottom-up proteomics. Despite early successes, shotgun proteomics of cyanobacteria remains technically challenging. This is, in part, due to a dense cell wall structure that prevents efficient protein extraction and a large amount of metabolites, pigments, and polysaccharides that interfere with LC–MS analysis. In a recent study, we have partially addressed these challenges by developing EXCRETE (enhanced exoproteome characterization by mass spectrometry), a fast, inexpensive workflow that enables a deep description of the cyanobacterial exoproteome (Russo et al. 2024). Here, we turn our attention to the endoproteome.

Current workflows for cyanobacterial endoproteomics rely on mechanical lysis (e.g. bead beating) in the presence of detergents, often followed by a precipitation step and downstream LC–MS analysis with data-dependent acquisition (DDA) (Srivastava et al. 2024). While cell lysis in the presence of detergents is often necessary to increase the detection of membrane proteins, protein precipitation can cause sample losses of up to 50% and is difficult to automate (Feist and Hummon 2015). In addition, data-independent acquisition (DIA) has gained widespread use owing to its accuracy, sensitivity, and selectivity on par with targeted proteomics but with a proteome wide coverage (Meier et al. 2020).

In this study, we offer an approach to cyanobacterial endoproteomics that enhances speed, throughput, and proteome coverage. Our results demonstrate that single-pot solid phase-enhanced sample preparation (SP3) cleanup can replace protein precipitation following bead beating and that a recently developed trifluoroacetic acid (TFA) lysis strategy (Doellinger et al. 2020; Abele et al. 2023) is suitable for cyanobacterial proteomics. When combined with library-free DIA, these workflows allowed the detection of 83% to 85% of all predicted proteins of *Synechococcus elongatus* PCC 7942 (hereafter Syn7942) with high quantitative reproducibility. Ultimately, our work offers cost- and time-effective workflows that have the potential to be broadly applied in cyanobacterial proteomics.

## Results

**Figure 1.**
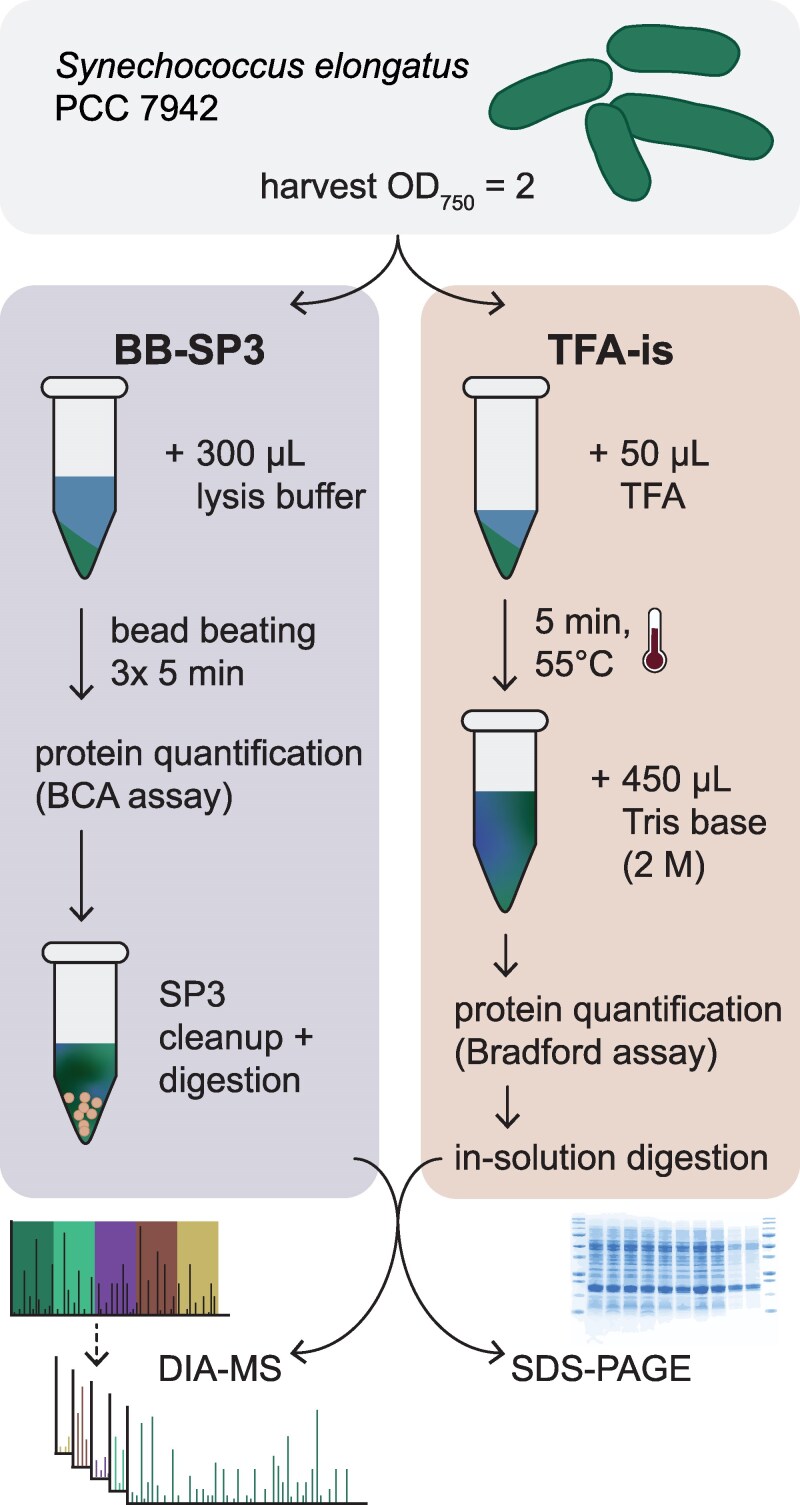
Schematic representation of the BB-SP3 and TFA-is DIA workflows. For each workflow, an optical density (OD)_750_ = 2 of a *Synechococcus elongatus* PCC 7942 culture was harvested. Following sample preparation, protein samples can be visualized by SDS–PAGE or digested to peptides for mass spectrometric analysis. BCA, bicinchoninic acid; SP3, single-pot solid phase-enhanced sample preparation; DIA–MS, data-independent acquisition mass spectrometry.

Our first approach coupled cell lysis, by bead beating in the presence of Triton X-100 and SDS (Russo et al. 2024), with SP3-based cleanup and protein digest (hereafter referred to as BB-SP3) (Fig. 1). SP3 protocols employ inexpensive magnetic beads to capture proteins in order to remove contaminants and perform enzymatic digestion (Hughes et al. 2014). With this we avoid protein precipitation steps or the use of expensive proprietary cleanup kits. Our second approach is based on cell lysis using 100% trifluoroacetic acid (TFA), followed by neutralization with Tris base and in-solution digestion (TFA-is, Fig. 1). This method was originally named SPEED and has recently been validated and optimized for bacterial cells (Doellinger et al. 2020; Abele et al. 2023). Two major advantages of the TFA-is workflow, in regard to BB-SP3, are the superior speed and throughput. We estimate that, with the TFA-is workflow, 96 samples can be processed up to the point of digestion in approximately 45 min. Whereas, with the BB-SP3 workflow, we estimate that the same number of samples would require 260 min, of which 190 min are needed for the low-throughput bead beating lysis step.

A departure from previous studies is that we used a data-independent rather than data-dependent method for acquisition (Fig. 1). In DDA, precursor ions are first detected in a survey scan, and the most intense ions are stochastically selected for fragmentation. DIA systematically fragments all ions within predefined *m/z* windows across the entire mass range, ensuring more comprehensive and reproducible peptide detection. The following results are based on the output of the directDIA + library-free workflow from the proprietary software Spectronaut. The data were also analyzed with the open-source DIA-NN (Demichev et al. 2020) and those results are briefly covered in the discussion.

### State-of-the-art workflows can identify 83% to 85% of the Syn7942 predicted proteins with high quantitative reproducibility

**Figure 2.**
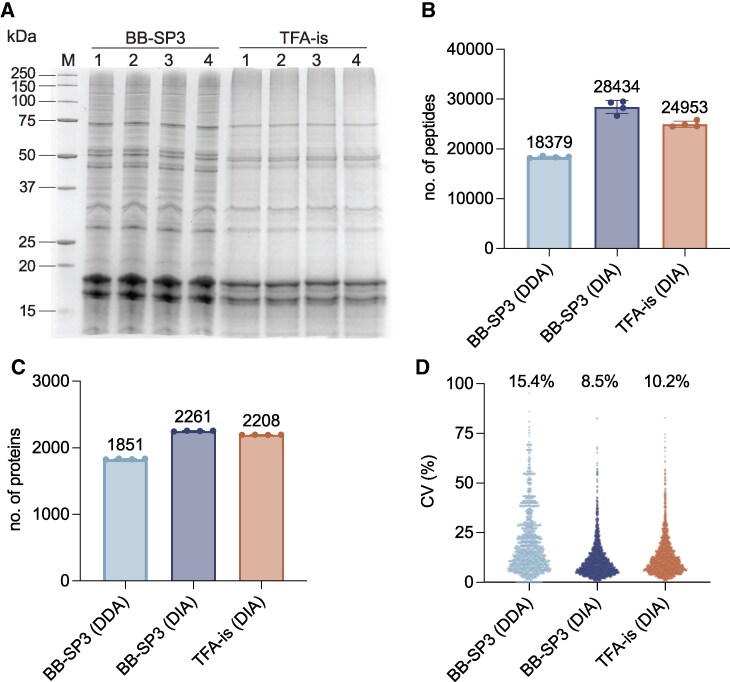
BB-SP3 and TFA-is workflows coupled to data-independent acquisition (DIA) analysis enable highly reproducible identification of 83% to 85% of Syn7942 predicted proteins in a single shotgun experiment. **A)** SDS–PAGE comparison of the BB-SP3 and TFA-is workflows. **B)** and **C)** Average number of peptides **(B)** and total number of proteins **(C)** identified by data-dependent acquisition (DDA) and DIA workflows. Dots represent biological replicates (*n* = 4), error bars represent standard deviation. **D)** Coefficient of variation (CV) of spectral counts (DDA) and protein intensities (DIA) across all four biological replicates for each workflow. Median CVs are shown above the bars.

To initially verify whether the BB-SP3 and TFA-is workflows were appropriate for cell lysis and protein extraction, we separated 15 *µ*g of total protein resulting from each workflow by SDS–PAGE (Fig. 2A). Visual inspection showed that both workflows produced qualitatively excellent intra-workflow reproducibility. Comparing both workflows, multiple bands appear stronger in the BB-SP3 samples (Fig. 2A). However, this is likely due to differences in protein amount estimation by the different assays rather than differential protein recovery levels. A comparison of DDA- and DIA-based LC–MS analyses of samples from the BB-SP3 workflow showed that a 60-min gradient in DDA acquisition mode (BB-SP3 DDA) identified, on average 18,379 peptides which corresponded to a total of 1,851 proteins and 70% of all predicted proteins (Fig. 2B, Supplementary Table S1). The BB-SP3 and TFA-is workflows with a 30-min gradient in DIA acquisition mode identified on average 28,434 and 24,593 peptides (Fig. 2B), respectively; an increase of 54% and 34% over DDA. This corresponded to a total of 2,261 and 2,208 proteins and 83% and 85% of all predicted proteins (Fig. 2C, Supplementary Tables S2 and S3). The numbers of identified proteins in this study is approximately 45% higher than recent shotgun proteomics studies of Syn7942 where a maximum of around 1,500 proteins were identified (Li et al. 2022; Singh et al. 2022; Sporre et al. 2023; Shi et al. 2024). However, these studies were done with older instruments which complicates direct comparisons. Analyzing the coefficients of variation (CVs) of the biological replicates showed that the BB-SP3 DDA workflow had a median CV of 15.4% while the BB-SP3 and TFA-is DIA workflows showed a higher quantitative reproducibility with median CVs of 8.5% and 10.2%, respectively (Fig. 2D). Given the superior performance of the DIA approach, we proceeded to take a closer look at the proteins identified by the BB-SP3 and TFA-is DIA workflows.

**Figure 3.**
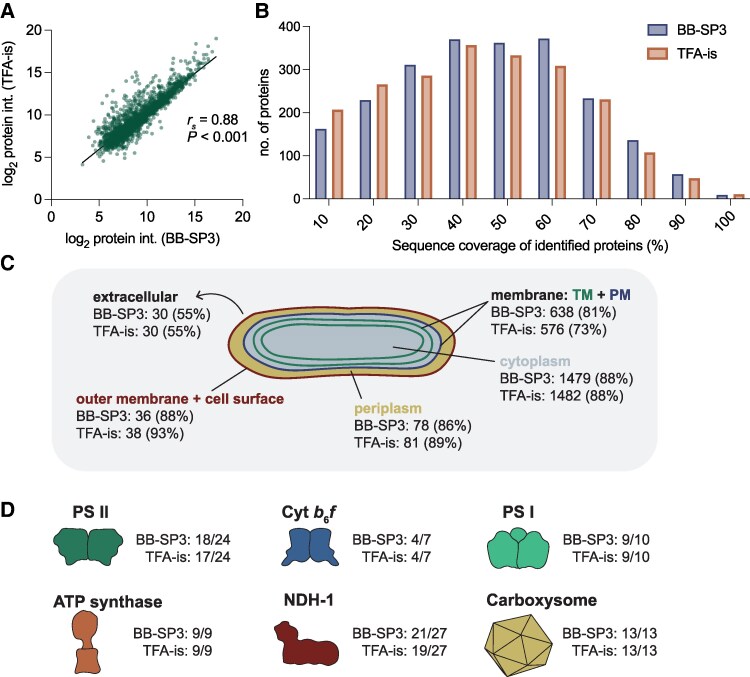
Global assessment of proteins identified by the BB-SP3 and TFA-is DIA workflows. **A)** Spearman correlation of log_2_ transformed protein intensities (int.). r_s_, Spearman's rank correlation coefficient. **B)** Frequency histogram depicting protein sequence coverage. Data are grouped into bins of 10%. **C)** Schematic representation of the predicted protein location. Numbers represent number of proteins identified. Percentages represent percentage of total proteins predicted per location. TM, thylakoid membrane; PM, plasma membrane. **D)** Schematic representation of the number of proteins identified from photosynthesis and carbon assimilation protein complexes. Depiction of protein complexes is not to scale.

A comparison of the intensity of the proteins identified by the BB-SP3 and TFA-is DIA workflows showed a significant inter-workflow correlation with a Spearman correlation coefficient of *r_s_* = 0.88, *P* < 0.001 (Fig. 3A). The average number of identified peptides per protein was 11 and 13, which led to an average protein sequence coverage of 44% and 46% for the BB-SP3 and TFA-is workflows, respectively (Fig. 3B). Analyzing the predicted location of the identified proteins we observed close to 90% of coverage for cytoplasmic, periplasmic and cell wall/outer membrane proteins in both workflows. The largest discrepancy was found in membrane proteins where an additional 8% of proteins with a predicted membrane location were identified with the BB-SP3 workflow. The lowest coverage for both workflows was observed for extracellular proteins. However, given this was an analysis of the endoproteome, this was expected (Fig. 3C).

Finally, to assess how well both workflows performed in identifying proteins involved in essential processes, we searched our dataset for proteins belonging to photosynthesis and carbon assimilation protein complexes. Remarkably, in a single shotgun experiment, we were able to identify the entirety of the ATP synthase and carboxysome complexes and the majority of the proteins belonging to the cytochrome *b*_6_*f*, NADH dehydrogenase-like (NDH-1), PSI, and PSII complexes (Fig. 3D, Supplementary Table S4). In this regard, BB-SP3 performed particularly well having identified an additional three low abundance membrane proteins: NdhC and NdhF4 in the NDH-1 complex, and PsbI in the PSII complex (Supplementary Table S4). Several proteins were not identified by either workflow (Supplementary Table S4). These can be grouped into (i) proteins that may not be expressed in our experimental conditions (e.g. NdhD3, NdhF3 in a CO_2_-enriched atmosphere) and (ii) small (<5 kDa) membrane proteins with single tryptic peptides that likely evade detection (e.g. PsbJ, PsbK, PetG, PetM).

### The BB-SP3 dataset is enriched in membrane proteins while the TFA-is dataset is enriched in small proteins

**Figure 4.**
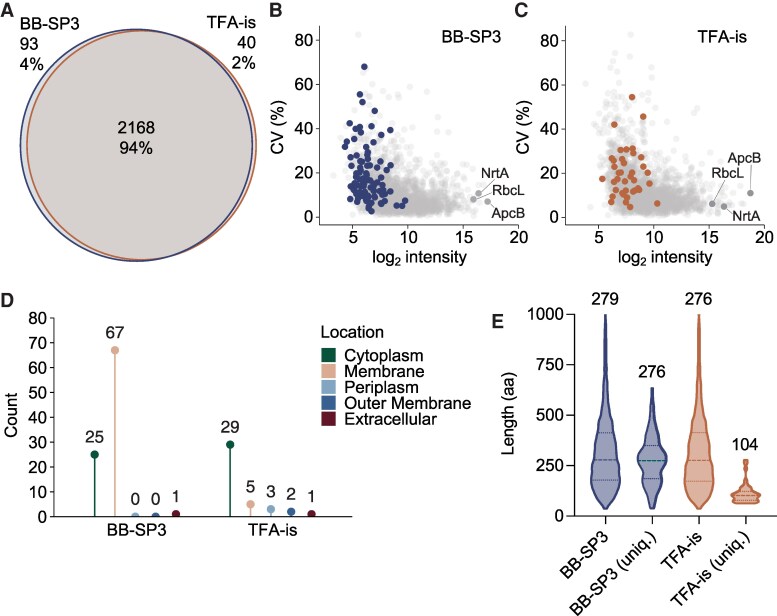
The BB-SP3 and TFA-is workflows have different strengths. **A)** Venn diagram representing the overlap of protein identifications with BB-SP3 and TFA-is. **B)** and **C)** Coefficient of variation (CV) of the raw protein intensities ordered by log_2_ protein intensity. Colored dots represent unique proteins. The position of the beta subunit of allophycocyanin ApcB, the nitrate/nitrite binding protein NrtA, and the large chain of ribulose bisphosphate carboxylase RbcL is marked. **D)** Distribution of unique proteins by cellular location and workflow. **E)** Comparison of length distribution of all identified proteins versus unique (uniq.) proteins for each workflow. Dashed lines represent the median, which is also shown numerically above the bars. Dotted lines represent the top and bottom quartiles. Only proteins up to 1,000 amino acids are depicted. Sample size (no. of proteins) left to right: 2,229; 93; 2,176; 40.

The analysis of photosynthesis and carbon assimilation protein complexes suggested that the BB-SP3 workflow may present a slight bias toward the identification of membrane proteins. This prompted us to investigate the set of unique proteins identified by each workflow. Of all the proteins identified, 2,168 (94%) were identified by both workflows, 93 (4%) were unique to BB-SP3, and 40 (2%) were unique to TFA-is (Fig. 4A). The most abundant proteins were identified by both workflows while the proteins that were unique to either workflow were all mid- to low-abundant (Fig. 4B and C). Of the 93 unique proteins found using the BB-SP3 workflow, 67 were membrane proteins. This represents an additional 8% of total proteins with a predicted membrane location (Fig. 4D, Supplementary Table S5). These include multiple membrane proteins of low abundance annotated as beneficial/essential (i.e. causing a growth defect or lethal when mutated, Rubin et al. 2015): the twin-arginine translocation (Tat) translocase TatC, the aforementioned NdhC (NDH-1 complex) and PsbI (PSII), the bicarbonate transporter SbtA, the lipoprotein signal peptidase LspA, proteins involved in lipopolysaccharide assembly (Synpcc7942_0175) and cobalt transport (Synpcc7942_2341), and plastoquinone (Synpcc7942_0152) and cobalamin (Synpcc7942_1441) biosynthesis. Amongst the 40 unique proteins found using the TFA-is workflow, several known proteins, such as the soluble electron carrier cytochrome c_6_, the bicarbonate transporter CmpD, and the prepilin leader peptidase PilD, were identified (Supplementary Table S6). Of the remaining proteins, the majority were cytoplasmic proteins of unknown function (Fig. 4D, Supplementary Table S6). One notable observation was that 19 out of the 40 unique proteins were small proteins (Fig. 4E). Small proteins are proteins of <100 amino acids (aa) that are typically too short for enzymatic activity but often have important structural and regulatory roles (Kraus and Hess 2025). The small proteins uniquely identified with the TFA-is workflow were generally poorly described. However, we identified several proteins annotated as essential (Rubin et al. 2015) such as a putative sulfur carrier (Synpcc7942_2075), two putative type II toxin-antitoxin systems (Synpcc7942_0871 and Sypncc7942_1209), and a putative ferredoxin (Synpcc7942_0814). The remaining nonessential small proteins were putatively annotated with diverse roles such as cell wall biogenesis, phosphate and copper transport, and stress tolerance. The majority of the unique proteins with more than 100 aa still remained under 150 aa (Fig. 4E, Supplementary Table S6). Overall, the TFA-is workflow identified an additional 7% of all genome-predicted small proteins.

## Discussion

In this study, we demonstrate that by streamlining LC–MS sample preparation and using a DIA approach we identified 83% to 85% of the Syn7942 proteome. Coupled with our recently developed EXCRETE workflow (Russo et al. 2024), we can now obtain in-depth descriptions of the endo- and exoproteome with inexpensive protocols that require only a couple of hours of hands-on time and can easily be adapted to a 96-well microplate format for high-throughput processing.

In one approach, we applied a recent TFA-is workflow to cyanobacterial samples. Lysis was done solely with the addition of 100% TFA and incubation at 55 °C for 5 min with no requirement for specialized equipment. For the BB-SP3 workflow, the use of an SP3 protocol to remove the lysis buffer contaminants constitutes a major improvement in comparison to widely used cleanup strategies and an entire 96-well microplate can be processed in 45 min. Using the BB-SP3 workflow we also compared data acquisition methods. While the number of proteins identified in DDA already surpassed the literature, the analysis of the same sample with DIA allowed for the identification of an additional 410 proteins. For the most abundant proteins, DIA and DDA exhibited a comparable performance. However, when it came to proteins of mid to lower abundance, the DDA dataset exhibited a large number of missing values which led to the lack of protein identification in sufficient replicates or an inflation of CVs for proteins that were confidently identified. The problem of missing values is ascribed to the stochastic nature of DDA and has been extensively discussed in the literature (Albrecht et al. 2010; Liu and Dongre 2021). Despite being widely used in proteomics studies, the lack of penetration of DIA methods in cyanobacterial research has been attributed to the absence of validated spectral libraries (Battchikova et al. 2018). However, computational advances have unlocked so-called “library-free” approaches which rely on *in silico* predicted libraries and strict false discovery rate control (Zhang et al. 2023). Here we showed that state-of-the-art DIA methods can efficiently generate *in silico* predicted libraries that are suitable for cyanobacterial proteomics solely using a predicted proteome file as input. The generation of *in silico* predicted libraries typically requires more computational resources and longer processing times. However, here, the smaller size of prokaryotic genomes is an advantage. As an example, all the samples in this study were processed by Spectronaut in 6 h. A reanalysis of our data with the open-source DIA-NN showed similar results to the Spectronaut output (BB-SP3: 2,234 proteins, *r_s_* = 0.90, *P* < 0.001; TFA-is: 2,151 proteins, *r_s_* = 0.89, *P* < 0.001; Supplementary Fig. S1, Supplementary Table S7) but only required 140 min processing time.

Previous to this study, efforts to increase proteome coverage of Syn7942 often included more laborious workflows. For example, label-free studies have typically benefited from some degree of cell fractionation, as exemplified by a recent study where 1,200 proteins were identified solely from isolated Syn7942 thylakoid membranes (Huokko et al. 2021). In the absence of fractionation, the use of label-based proteomics has enabled the identification of low abundance proteins. For example, a pioneering study using tandem mass tag (TMT) labeling and peptide fractionation identified 2,179 proteins (82% proteome coverage) with SWATH-DIA supported by a DDA-generated spectral library (Guerreiro et al. 2014). In another study, quantitative conCATamer-based MS enabled the determination of the absolute stoichiometric composition of the entire Syn7942 β-carboxysome (Sun et al. 2024). In our work, 87% of the predicted proteome was identified between both label-free DIA workflows. This is likely approaching the limit of the proteins produced under these conditions. While expression of 87% of all open reading frames is quite high, this supports the idea that Syn7942 has a small, streamlined, genome with little redundancy (Rubin et al. 2015). For more complex cyanobacteria, such as *Synechocystis* sp. PCC 6803 and *Nostoc* spp., the number of identified proteins in shotgun studies varies between 55% and 65% of the predicted proteome (Russo et al. 2024; Srivastava et al. 2024; Teikari et al. 2025). While this lower percentage of identified proteins may reflect higher levels of proteomic plasticity, it is likely that improvements in sample preparation and data collection can push this further. Higher proteome coverage has only been achieved with proteogenomic workflows where proteome profiling under various cultivation conditions is combined with genome re-annotation (Yang et al. 2014; Yu et al. 2021; Spät et al. 2023). Combined with our workflows, this approach could extend Syn7942 proteome coverage beyond the 87% achieved in this study.

In conclusion, detergent-based sample preparation workflows are worth considering for specific applications, such as the identification of membrane proteins. However, the TFA-is workflow is a faster, more cost-effective, sample preparation approach that should be suitable for most proteomics studies, and in particular for the identification of small proteins. Ultimately, our study shows that, by streamlining sample preparation and adopting state-of-art analysis technologies, we can push the limit of cyanobacterial proteomics.

## Materials and methods

### Cultivation

*S. elongatus* PCC 7942 (obtained from the Pasteur Culture Collection of Cyanobacteria) was maintained on BG-11 medium supplemented with 5 mm 4-(2-hydroxyethyl)-1-piperazineethanesulfonic acid (HEPES) pH 7.5 (BG-11_H_ medium) and 1.5% (w/v) Kobe I agar (Carl Roth) at 30 °C with continuous illumination of approximately 25 *μ*mol photons m^−2^ s^−1^. Liquid cultures were grown in BG-11_H_ at 30 °C with continuous illumination of 50 *μ*mol photons m^−2^ s^−1^ and bubbled with 3% (v/v) CO_2_-supplemented air. For the proteomics experiments, cultures were initially inoculated from agar plates and grown for 3 days. The preculture was then washed with BG-11_H_, and the main cultures were inoculated at an optical density at 750 nm (OD_750_) = 0.4 and grown for 4 days.

### BB-SP3 workflow

Endoproteome fractions were collected by centrifuging an OD_750_ equivalent of 2 (approximately 300 *µ*L cell culture) for 10 min at 10,000 × *g*. Cell lysis was then done according to (Russo et al. 2024) but sodium deoxycholate was omitted from the lysis buffer. Protein concentrations were determined by a Pierce BCA assay (Thermo Fisher) and 20 *µ*g total protein were reduced with 10 mm tris(2-carboxyethyl)phosphine (TCEP) and alkylated with 40 mm chloroacetamide (CAA) at 95 °C for 5 min. For the SP3 cleanup, the volume of all samples was normalized to 25 *µ*L and NaCl and SDS were added to a final concentration of 10 mm and 1%, respectively. SiMAG-Carboxyl magnetic particles (chemicell GmbH, #1201) were then added to a concentration of 0.5 *µ*g µL^−1^, one volume of EtOH was added to a final concentration of 50% (v/v) EtOH, and samples were incubated for 10 min on a thermoshaker at 25 °C and 750 rpm. The magnetic particles were then separated on a magnetic rack for 1 min. Supernatants were removed and the samples were washed, on magnet, three times with 80% (v/v) EtOH. Following the final wash, samples were removed from the magnetic rack and air dried for 10 min. The magnetic particles were then carefully resuspended in 100 *µ*L 25 mm ammonium bicarbonate with 0.5 *µ*g MS grade Trypsin/LysC (Promega) (enzyme/protein ratio of 1:40 (w/w)) for overnight protein digestion. Following digestion, the magnetic particles were separated and the supernatants recovered. Subsequent peptide purification and desalting was done according to (Russo et al. 2024).

### Trifluoroacetic acid lysis with in-solution protein digest (TFA-is) workflow

Endoproteome fractions were obtained as in the BB-SP3 workflow but then resuspended in 50 *μ*L 100% trifluoroacetic acid (TFA). The samples were incubated for 5 min at 55 °C and then neutralized with 450 *μ*l 2 m Tris base (pH not adjusted). The pH after neutralization was approximately 8.2. Protein concentrations were determined by a Bradford assay (Pierce Detergent Compatible Bradford Assay, #23246) and 20 *µ*g total protein were reduced with 10 mm TCEP and alkylated with 40 mm CAA at 95 °C for 5 min. Samples were diluted with one volume of water to a final concentration of 1 m Tris base and 5% TFA. For overnight protein digestion, 0.5 *µ*g MS grade Trypsin/LysC (Promega) (enzyme/protein ratio of 1:40 (w/w)) were then added. Subsequent peptide purification and desalting was done according to (Russo et al. 2024) with the exception that the pH of the peptide mixture had to be further adjusted by the addition of 3 *µ*L 100% TFA.

### SDS–PAGE analysis

Following protein quantification, 15 *µ*g protein per sample was incubated for 5 min at 95 °C with sample loading buffer prior to separation by SDS–PAGE. Proteins were separated on a 4% to 12% Criterion XT Bis-Tris gel (Bio-Rad) in XT MOPS running buffer at 200 V.

### LC–MS analysis

Purified and desalted peptides were analyzed using nanoflow reversed-phase LC (Bruker nanoElute) coupled to a trapped ion mobility spectrometry quadrupole time-of-flight mass spectrometer (Bruker timsTOF HT). Peptides were separated on a 15 cm × 75 *μ*m column packed in-house with 1.9 *μ*m C_18_ particles (ReproSil-Pur 120 C18-AQ, Dr. Maisch). For both methods, the binary mobile phase consisted of solvent A (0.1% (v/v) formic acid in water) and solvent B (0.1% (v/v) formic acid in acetonitrile), and the flow rate was set at 0.5 *μ*L min^−1^. For DDA, a 60-min gradient was used. The gradient started with a linear increase from 2% to 35% B over 52 min, followed by an increase to 95% B within 5 min, and was held for an additional 3 min at 95% B. For DIA, a 30-min gradient was used. The gradient started with a linear increase from 0% to 30% B over 27 min, followed by an increase to 95% B within 1 min and was held for an additional 2 min at 95% B. Electrospray ionization was performed using a CaptiveSpray source (Bruker Daltonics) from a pulled emitter tip. *Data acquisition parameters* and *Raw data processing* are described in Supplementary Methods.

### Bioinformatic and statistical analysis

All experiments were performed in four biological replicates. A protein group was considered identified when it was present in a minimum of three replicates. Proteins were annotated using DeepLocPro 1.0 (Moreno et al. 2024) and UniProtKB (The UniProt Consortium 2023). Data analysis and visualization were performed using custom scripts in R (4.4.2), with packages dplyr (1.1.4) and tidyplots (0.2.1) (Engler 2025), and GraphPad Prism (10.4.1).

### Accession numbers

Proteomics data generated during this study are available via PRIDE with the identifier PXD062851. Accession numbers of the proteins identified in this study are summarized in Supplementary Tables S2 to S7.

## Supplementary Material

kiaf334_Supplementary_Data

## Data Availability

The raw MS data and associated tables (i.e. peptide lists, unfiltered protein groups) have been deposited to the ProteomeXchange Consortium via the PRIDE partner repository and are publicly available with the identifier PXD062851. Code to reproduce Figs. 3B and 4B, 4C and 4D is available at Zenodo DOI: https://doi.org/10.5281/zenodo.15234135. All other data are available from the corresponding author on reasonable request.

## Supplementary data

The following materials are available in the online version of this article.

**Supplementary Figure S1.** Comparison of proteins identified by Spectronaut and DIA-NN with both workflows by Spearman correlation. A) and B) Correlation of log_2_ transformed protein intensities (int.) for the (A) BB-SP3 and (B) TFA-is workflows. *r_s_* = Spearman's rank correlation coefficient.

**Supplementary Table S1.** List of proteins identified with the BB-SP3 DDA workflow.

**Supplementary Table S2.** List of proteins identified with the BB-SP3 DIA workflow.

**Supplementary Table S3.** List of proteins identified with the TFA-is DIA workflow.

**Supplementary Table S4.** List of identified proteins belonging to photosynthesis and carbon assimilation protein complexes.

**Supplementary Table S5.** Unique proteins identified with the BB-SP3 DIA workflow.

**Supplementary Table S6.** Unique proteins identified with the TFA-is DIA workflow.

**Supplementary Table S7.** List of proteins identified by DIA-NN with the BB-SP3 and TFA-is DIA workflows.

**Supplementary Methods.** LC–MS data acquisition parameters; Raw data processing

## Dive Curated Terms

The following phenotypic, genotypic, and functional terms are of significance to the work described in this paper:

TFA CHEBI: CHEBI:45892

CAA CHEBI: CHEBI:234507

TCEP CHEBI: CHEBI:63213

proteins CHEBI: CHEBI:36080
