# Supplementary material for: Library-free data-independent acquisition mass spectrometry enables comprehensive coverage of the cyanobacterial proteome
Source: Plant Physiol. 2025 Aug 12;199(1):kiaf334. doi: 10.1093/plphys/kiaf334 (PMC12415867; doi:10.1093/plphys/kiaf334)
Supplement: kiaf334_Supplementary_Data [file kiaf334_supplementary_data.zip › Supplementary Table list.docx]

Supplementary Table S1. List of proteins identified with the BB-SP3 DDA workflow.

Supplementary Table S2. List of proteins identified with the BB-SP3 DIA workflow.

Supplementary Table S3. List of proteins identified with the TFA-is DIA workflow.

Supplementary Table S4. List of identified proteins belonging to photosynthesis and carbon assimilation protein complexes.

Supplementary Table S5. Unique proteins identified with the BB-SP3 DIA workflow.

Supplementary Table S6. Unique proteins identified with the TFA-is DIA workflow.

Supplementary Table S7. List of proteins identified by DIA-NN with the BB-SP3 and TFA-is DIA workflows.
