# Supplementary material for: Library-free data-independent acquisition mass spectrometry enables comprehensive coverage of the cyanobacterial proteome
Source: Plant Physiol. 2025 Aug 12;199(1):kiaf334. doi: 10.1093/plphys/kiaf334 (PMC12415867; doi:10.1093/plphys/kiaf334)
Supplement: kiaf334_Supplementary_Data [file kiaf334_supplementary_data.zip › DIAΓÇôMS for cyanobacterial proteomics supp figures.pdf]

Supplementary Figures

Library-free data-independent acquisition mass spectrometry enables comprehensive coverage of the cyanobacterial proteome

David A. Russo[1], Felix R. Schneidmadel[2] and Julie A. Z. Zedler[3*]*

[1] Bioorganic Analytics, Institute for Inorganic and Analytical Chemistry, Friedrich Schiller University Jena, 07743 Jena, Germany

[2] Functional Proteomics, Jena University Hospital, 07747 Jena, Germany

[3] Synthetic Biology of Photosynthetic Organisms, Matthias Schleiden Institute for Genetics, Bioinformatics and Molecular Botany, Friedrich Schiller University Jena, 07743 Jena, Germany

[*]Corresponding authors:
Email: david.russo@uni-jena.de, julie.zedler@uni-jena.de

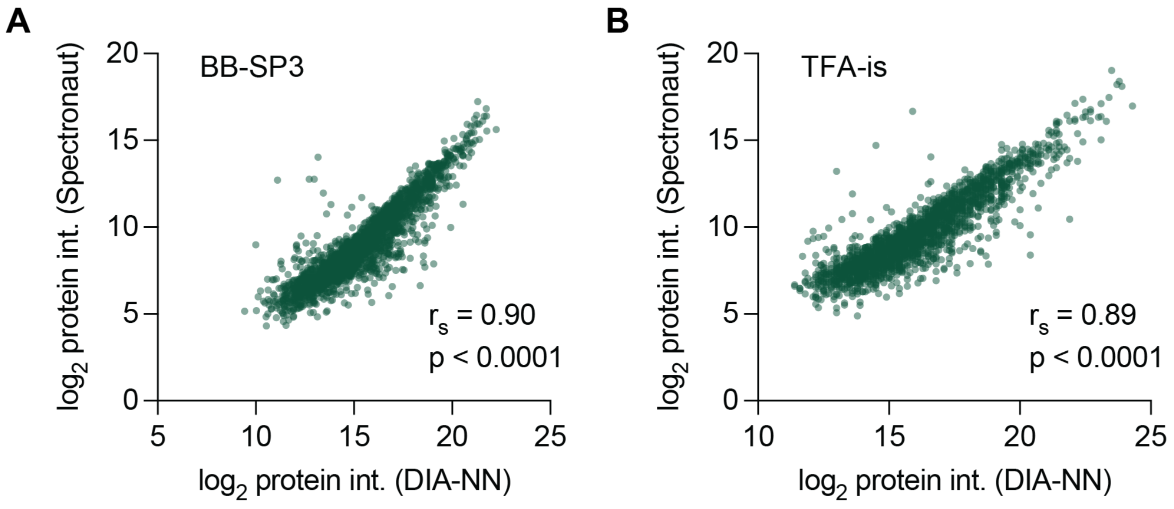

Supplementary Figure S1. Comparison of proteins identified by Spectronaut and DIA-NN with both workflows by Spearman correlation. A) and B) Correlation of log_2 transformed protein intensities (int.) for the (A) BB-SP3 and (B) TFA-is workflows. r_s, Spearman's rank correlation coefficient.
