# Supplementary material for: Library-free data-independent acquisition mass spectrometry enables comprehensive coverage of the cyanobacterial proteome
Source: Plant Physiol. 2025 Aug 12;199(1):kiaf334. doi: 10.1093/plphys/kiaf334 (PMC12415867; doi:10.1093/plphys/kiaf334)
Supplement: kiaf334_Supplementary_Data [file kiaf334_supplementary_data.zip › DIAΓÇôMS for cyanobacterial proteomics supp methods.pdf]

Supplementary Methods

Library-free data-independent acquisition mass spectrometry enables comprehensive coverage of the cyanobacterial proteome

David A. Russo[1], Felix R. Schneidmadel[2] and Julie A. Z. Zedler[3*]*

[1] Bioorganic Analytics, Institute for Inorganic and Analytical Chemistry, Friedrich Schiller University Jena, 07743 Jena, Germany
[2] Functional Proteomics, Jena University Hospital, 07747 Jena, Germany
[3] Synthetic Biology of Photosynthetic Organisms, Matthias Schleiden Institute for Genetics, Bioinformatics and Molecular Botany, Friedrich Schiller University Jena, 07743 Jena, Germany

[*]Corresponding authors:
Email: david.russo@uni-jena.de, julie.zedler@uni-jena.de

Table of Contents

Supplementary Methods 2
LC-MS data acquisition parameters 2
Raw data processing 2
References 3

Supplementary Methods

LC-MS data acquisition parameters

For DDA, precursor ions were selected for fragmentation based on their position in the $1/K_0$ versus *m/z* space, with a range of $1/K_0$ = 0.6 to 1.5 Vs cm^{-2}, an *m/z* range of 100 to 1700, and accumulation and ramp times of 100 ms. Dynamic exclusion was applied with an exclusion time of 0.4 min once the target intensity threshold of 20,000 a.u. was reached. The quadrupole isolation window was set to 2 Th for m/z < 700 and 3 Th for m/z > 700. Instrument calibration was performed using ions from the Agilent ESI LC/MS tuning mix, with *m/z* values of 322.0481, 622.0289, 922.0097, and 1221.9906 and corresponding $1/K_0$ values of 0.7319 Vs cm^{-2}, 0.9848 Vs cm^{-2}, 1.1895 Vs cm^{-2}, and 1.3820 Vs cm^{-2}. All DIA measurements were performed using the diaPASEF acquisition mode (Meier et al., 2020) on a trapped ion mobility spectrometry (TIMS) quadrupole time-of-flight mass spectrometer. The instrument was operated with a predefined precursor isolation scheme in both the *m/z* and ion mobility dimensions, utilizing equidistant 25 *m/z*-wide windows. The MS1 scan range was set to 100–1700 *m/z*, and the ion mobility range was $1/K_0$ = 0.60–1.60 Vs cm^{-2}. Each diaPASEF cycle lasted 1.8 seconds and consisted of 8 scans, cumulatively covering sixteen 25 *m/z* windows. MS2 spectra were acquired in the range of 400–1201 Da and $1/K_0$ = 0.60–1.43 Vs cm^{-2} for the ion mobility. The ion accumulation and ramp times in the dual TIMS analyzer were both set to 100 ms. Collision energy was applied in a linearly decreasing gradient, ranging from 59 eV at $1/K_0$ = 1.4 Vs cm^{-2} to 20 eV at $1/K_0$ = 0.6 Vs cm^{-2}.

Raw data processing

DDA raw files were processed using FragPipe (20.0), integrating MSFragger (3.8), IonQuant (1.9.8), and Philosopher (5.1.0) (Yu et al., 2020). Data analysis followed the "Default" workflow for "IM-MS (Ion Mobility, timsTOF only)". The protein search database comprised 2657 proteins from Syn7942 (Uniprot: UP000889800, accessed December 2024), along with 2778 decoy sequences and potential contaminant proteins incorporated by FragPipe. *In silico* peptides were derived using the strict trypsin digestion rule for trypsin and allowing up to two missed cleavages within a mass range of 500-5000 Da and a peptide length of 7-50 amino acids. Cys carbamidomethylation was included as a fixed modification and Met oxidation and N-terminal acetylation as variable modifications. Peptide spectrum matching was performed with an absolute precursor mass tolerance of 20 ppm and a fragment mass tolerance of 20 ppm. DIA raw files were processed in Spectronaut v19.6 (Biognosys) (Bruderer et al., 2015) using the directDIA+ workflow with default parameters. False discovery rate (FDR) control was applied at both precursor and protein levels at <1% using a target-decoy approach. Cys carbamidomethylation was included as a fixed modification and Met oxidation and N-terminal acetylation as variable modifications. Spectral matching was performed against the Syn7942 reference proteome (Uniprot: UP000889800, accessed December 2024). DIA raw files were also analyzed using DIA-NN version 2.1 (Demichev et al., 2020) with default settings in library-free mode. The same Syn7942 FASTA file (Uniprot: UP000889800, accessed December 2024) as used for FragPipe and Spectronaut was provided as input for library prediction. Deep learning-based prediction of spectra, retention times, and ion mobilities was enabled. Trypsin/P was specified as the protease, allowing up to two missed cleavages. Met oxidation (Ox[M]) was set as a fixed modification. Match-between-runs was enabled across all samples to improve data completeness. A precursor-level FDR of 1% was applied.

References

Bruderer R, Bernhardt OM, Gandhi T, Miladinović SM, Cheng L-Y, Messner S, Ehrenberger T, Zanotelli V, Butscheid Y, Escher C, Vitek O, Rinner O, Reiter L. Extending the Limits of Quantitative Proteome Profiling with Data-Independent Acquisition and Application to Acetaminophen-Treated Three-Dimensional Liver Microtissues. *Mol Cell Proteomics* 2015:14(5), 1400–1410. https://doi.org/10.1074/mcp.M114.044305

Demichev V, Messner CB, Vernardis SI, Lilley KS, Ralser M. DIA-NN: neural networks and interference correction enable deep proteome coverage in high throughput. *Nat Methods* 2020:17, 41–44. https://doi.org/10.1038/s41592-019-0638-x

Meier F, Brunner A-D, Frank M, Ha A, Bludau I, Voytik E, Kaspar-Schoenefeld S, Lubeck M, Raether O, Bache N, Aebersold R, Collins BC, Röst HL, Mann M. diaPASEF: parallel accumulation–serial fragmentation combined with data-independent acquisition. Nat Methods 2020:17, 1229–1236. https://doi.org/10.1038/s41592-020-00998-0

Yu F, Haynes SE, Teo GC, Avtonomov DM, Polasky DA, Nesvizhskii AI. Fast Quantitative Analysis of timsTOF PASEF Data with MSFragger and IonQuant. *Mol Cell Proteomics* 2020:19(9), 1575–1585. https://doi.org/10.1074/mcp.TIR120.002048
